# Gain of chromosome 21 increases the propensity for *P2RY8:* *:CRLF2* acute lymphoblastic leukemia *via* increased *HMGN1* expression

**DOI:** 10.3389/fonc.2023.1177871

**Published:** 2023-07-06

**Authors:** Elyse C. Page, Susan L. Heatley, Jacqueline Rehn, Paul Q. Thomas, David T. Yeung, Deborah L. White

**Affiliations:** ^1^ Blood Cancer Program, Precision Cancer Medicine Theme, South Australian Health and Medical Research Institute, Adelaide, SA, Australia; ^2^ School of Biological Sciences, Faculty of Sciences, Engineering, and Technology, University of Adelaide, Adelaide, SA, Australia; ^3^ Adelaide Medical School, Faculty of Health and Medical Science, University of Adelaide, Adelaide, SA, Australia; ^4^ SA Gene Editing Program, Lifelong Health Theme, South Australian Health and Medical Research Institute, Adelaide, SA, Australia; ^5^ Australasian Leukaemia and Lymphoma Group, Melbourne, VIC, Australia; ^6^ Department of Hematology, Royal Adelaide Hospital and SA Pathology, Adelaide, SA, Australia; ^7^ School of Pharmacy and Medical Science, University of South Australia, Adelaide, SA, Australia; ^8^ Australian and New Zealand Children’s Hematology/Oncology Group (ANZCHOG), Clayton, VIC, Australia

**Keywords:** leukemia, gene expression, *HMGN1*, *P2RY8::CRLF2*, CRISPR/Cas9

## Abstract

Acute lymphoblastic leukemia (ALL) patients with a gain of chromosome 21, intrachromosomal amplification of chromosome 21 (iAMP21), or Down syndrome (DS), have increased expression of genes in the DS critical region (DSCR) of chromosome 21, including the high-mobility group nucleosome-binding protein 1, *HMGN1*. Children with DS are predisposed to develop hematologic malignancies, providing insight into the role of chromosome 21 in the development of leukemias. A 320-kb deletion in the pseudoautosomal region of the X/Y chromosome in leukemic cells, resulting in a gene fusion between the purinergic receptor and cytokine receptor-like factor-2 (*P2Y Receptor Family Member 8 (P2RY8)::CRLF2*), is a common feature in ~60% of DS-ALL and ~40% of iAMP21 patients, suggesting a link between chromosome 21 and *P2RY8::CRLF2*. In an Australian cohort of pediatric B-ALL patients with *P2RY8::CRLF2* (*n* = 38), eight patients harbored gain of chromosome 21 (+21), and two patients had iAMP21, resulting in a significantly increased *HMGN1* expression. An inducible CRISPR/Cas9 system was used to model *P2RY8::CRLF2* and investigate its cooperation with *HMGN1*. This model was then used to validate *HMGN1* as an influencing factor for *P2RY8::CRLF2* development. Using Cas9 to cleave the DNA at the pseudoautosomal region without directed repair, cells expressing *HMGN1* favored repair, resulting in *P2RY8::CRLF2* generation, compared with cells without *HMGN1*. CRISPR/Cas9 *P2RY8::CRLF2* cells expressing *HMGN1* exhibit increased proliferation, thymic stromal lymphopoietin receptor (TSLPR) expression, and JAK/STAT signaling, consistent with cells from patients with *P2RY8::CRLF2*. Our patient expression data and unique CRISPR/Cas9 modeling, when taken together, suggest that *HMGN1* increases the propensity for *P2RY8::CRLF2* development. This has important implications for patients with DS, +21, or iAMP21.

## 1 Introduction

Gain of chromosome 21 is the most common whole chromosome copy number variation (CNV) that occurs in hematological malignancies (1, 2), with the highest frequency in acute lymphoblastic leukemia (ALL) at ~15% (3). Children with Down syndrome (DS) and constitutional trisomy 21 have a 20-fold increased risk of developing ALL (4), a 150-fold increased risk of developing acute myeloid leukemia (AML), and are 400–600 times more likely to develop acute megakaryoblastic leukemia (AMKL) (5, 6). The purinergic receptor and cytokine receptor-like factor-2 (*P2Y Receptor Family Member 8 (P2RY8)::CRLF2*) gene fusion has been identified in ~60% of DS-ALL (+21) patients and ~40% of patients with intrachromosomal amplification of chromosome 21 (iAMP21), compared with only 5%–16% of pediatric ALL patients without +21 (7, 8). The genomic basis for the predisposition in DS-ALL has been investigated (9), but the role of chromosome 21 remains unknown.

Chromosome 21 harbors over 30 candidate genes that may contribute to leukemogenesis (10), including the high-mobility group nucleosome-binding protein 1 (*HMGN1*) (10). Many genes in the Down syndrome critical region (DSCR) of chromosome 21 have been implicated in various hematological malignancies due to their roles in cancer-associated or gene activation pathways (10, 11). These genes have not yet been linked with the high proportion of DS-ALL patients with *P2RY8::CRLF2*. Genes including the dual-specificity tyrosine phosphorylated and regulated kinase 1A (*DYRK1A*), ETS-related gene (*ERG*), ETS variant transcription factor 6 (*ETV6*), EBF transcription factor 1 (*EBF1*), and RUNX family transcription factor 1 (*RUNX1*) have been studied in ALL (12–18), while many more have been characterized in the context of AML, including the GATA-binding factor 1 (*GATA1*) (2), ubiquitin-specific peptidase 16 (*USP16*) (19), chromatin assembly factor 1 (*CHAF1B*) (20), and the microRNAs (mir99A and mir125b) (21, 22).

Interest in the potential involvement of *HMGN1* in leukemia development has arisen due to its demethylase activity associated with enhanced transcriptional activation (11). While ALL patients with *CRLF2* alterations are considered at high risk of treatment failure, there are no current effective targeted therapies for this cohort (23). CRISPR/Cas9 facilitates the modeling of individual patient genomic variants (24) with reduced off-target effects and higher efficacy than older gene editing technologies (25) and has been used to model AML fusions (26), but not ALL fusion genes. As there are no cell lines endogenously expressing *P2RY8::CRLF2*, it is necessary to establish a pre-leukemic cell model to investigate *HMGN1* and its effect on *P2RY8::CRLF2* fusion generation. We hypothesize that the expression of *HMGN1* prior to a Cas9-induced DNA break will lead to increased *P2RY8::CRLF2* development in cell lines compared with cells that have low *HMGN1* expression.

## 2 Methods

### 2.1 Cell lines and maintenance

HEK293T cells (ATCC, Manassas, VA, USA) were maintained in Dulbecco’s modified Eagle’s medium (MEM) and utilized for lentiviral transduction, and Jurkat cells (ATCC, Manassas, VA, USA), which were maintained in Roswell Park Memorial Institute (RPMI), were supplemented with 10% fetal calf serum (FCS), 200 mM of L-glutamine (SAFC Biosciences), 5,000 U/mL of penicillin, and 5,000 µg/mL of streptomycin sulfate.

### 2.2 Constructing the FgH1tUTG gRNA vector

The Benchling gRNA design tool (Biology Software, 2019, https://benchling.com) was used to design gRNAs targeting the intron following the first non-coding exon of *P2RY8* and preceding the first exon of *CRLF2* with 5′ *Esp*3I restriction sites (Key Resources Table). The FUCas9Cherry and FgH1tUTG plasmids were a gift from Marco Herold (Addgene #70182 and #70183) (27). The FgH1tUTG vector was digested with *Esp*3I [New England Biolabs (NEB) #R0734L] and rSAP (NEB #M0371L) for 1 h at 37°C. The complementary gRNAs were phosphorylated at a final concentration of 10 µM using T4 PNK (NEB #M0201L) and then diluted 1:125 with nuclease-free water. Moreover, 5 ng/µL of FgH1tUTG vector was digested with 0.8 pmol of diluted gRNA and ligated with T4 ligase overnight (NEB #M0204L) at 4°C. The ligation was transformed overnight into competent DH5α *E. coli* (NEB #C2987H) on ampicillin-containing Luria–Bertani agar plates. Single colonies were isolated and cultured for plasmid purification using the Qiagen QIAprep spin miniprep kit (#27104).

### 2.3 Lentiviral transduction

Lentivirus was produced by transfecting 5.5 µg of the FuCas9mCherry vector or FgH1tUTG gRNA vector, with packaging constructs pMD2.G (2.25 µg), pMDL-PRRE (3.375 µg), and pRSV-REV (1.575 µg), with 30 µL of lipofectamine added into 1 × 10^6^ HEK293T cells in a T25 culture flask in 5-mL media. Viral supernatant was harvested 48 h later and passed through a 0.45-µm filter. Jurkat cells at a concentration of 5 × 10^5^/mL were centrifuged at 1,800 rpm for 1 h with 30 µg/mL of polybrene in 4 mL of viral supernatant in a six-well plate (28).

### 2.4 Flow cytometry cell sorting

Jurkat cells transduced with FuCas9mCherry and FgH1tUTG were resuspended in 1 mL of RPMI with 2% FCS at a concentration of 1 × 10^7^ cells. This suspension was sorted on a BD FACSAria™ for GFP and mCherry double-positive cells. Pure populations were resuspended in 1 mL of RPMI with 2% FCS and sorted into single cells in a 96-well plate with 100 µL of RPMI with 20% FCS on a BD FACS Melody™. The clones were subcultured into 1 mL of media in a 24-well plate 3 weeks after sorting.

### 2.5 Genome-targeting efficiency assay

Jurkat cells transduced with Cas9 and gRNA vectors (Key Resources Table) were exposed to 1 µg/mL of doxycycline hyclate (Sigma-Aldrich, St. Louis, MO, USA) in milli-Q water for 72 h to induce the 320-kb deletion. gDNA was isolated from transduced cells by phenol-chloroform extraction, and the *P2RY8::CRLF2* fusion breakpoint was amplified *via* PCR using the Phusion kit (NEB, Notting Hill, VIC, Australia). The primer sequences are outlined in the Key Resources Table. Heteroduplexes were formed by denaturing the PCR products at 95°C for 5 min and reannealing the breakpoint amplification PCR product by slowly ramping down the temperature to room temperature. The reannealed PCR products were digested with 1 µL of T7 endonuclease I (NEB) for 1 h at 37°C. The resulting products were gel-purified (Qiagen, Venlo, The Netherlands) and Sanger-sequenced.

### 2.6 Surface flow cytometry

Transduced Jurkat cells were stained with 5 µL of TSLPR-APC or isotype control IgG2a (Invitrogen, Carlsbad, CA, USA) for 30 min in 100 µL of RPMI with 10% FCS on ice. Approximately 5 × 10^6^ cells were washed with 1 mL of RPMI with 10% FCS and resuspended in 200 µL of 1× PBS and read on a BD FACS Fortessa™ analyzer.

### 2.7 Intracellular flow cytometry

Jurkat cells were fixed with a final concentration of 1.6% paraformaldehyde for 10 min, washed in 1× PBS, and then permeabilized with 80% methanol overnight at -80°C (28). The cells were washed in 1× PBS followed by 1× PBS containing 1% bovine serum albumin (BSA). All intracellular staining procedures were carried out in the dark, on ice, for 60 min at room temperature in 1× PBS/1% BSA with the antibodies outlined in the Key Resources Table. The cells were washed in 1× PBS before reading on a BD FACSCanto™ analyzer.

### 2.8 Real-time PCR analysis

RNA was isolated from transduced Jurkat cells using TRIzol^®^ (Invitrogen), and cDNA was synthesized using Quantitect reverse transcriptase (Qiagen). SYBR Green reagents (Qiagen) were used with 10 µM of *CRLF2* or *HMGN1* primers as outlined in the Key Resources Table.

### 2.9 Proliferation assay

Jurkat cells were seeded at 390 cells/mL in duplicate in a 24-well plate. On days 0, 2, 4, and 6, 20 µL of CellTiter-Glo 2.0® reagent (Promega, Fitchburg, WI, USA) was added to 20 µL of cell suspension. Following 30 min of incubation in the dark, luminescence was measured on a Perkin Elmer Victor X5 luminometer set to luminescence at 0.1 s.

### 2.10 Development of a patient-derived xenograft murine model

NOD.Cg-Prkdc^scid^Il2rg^tm1Wjl^/SzJ (NSG) mice (The Jackson Laboratory, Bar Harbor, ME, USA) were treated subcutaneously with 0.1 mg of Baytril in 0.9% sodium chloride per 10 g body weight prior to sublethal gamma irradiation at 200 cGy. Spleen- or bone marrow (BM)-derived ALL patient (*P2RY8::CRLF2* or *BCR::ABL1*) blasts (1 × 10^6^ cells) were injected into the tail vein of NSG mice. Engraftment and disease progression were monitored by fortnightly blood sampling from the tail vein and flow cytometric analysis of hCD45+. The animals were monitored daily and were humanely killed when they displayed clinical signs of leukemia (e.g., weight loss, reduced activity, ruffled fur). At the end of the experiment, cardiac bleeding and complete blood count (CBC) were performed, and the spleen, liver, and BM were harvested. Flow cytometric immunophenotyping was performed on peripheral blood and/or BM (28). All experiments were performed on protocols approved by the Institutional Animal Ethics Committee.

### 2.11 mRNA sequencing

mRNA sequencing was performed on the blast cells of pediatric and adolescent/young adult ALL patients (*n* = 508) using the Universal Plus mRNA seq with NuQuant kit as per the manufacturer’s instructions, with 1 µg of high-quality total RNA, and sequenced on the Illumina NextSeq 500 platforms (29). A read depth of 70 million reads was achieved for most samples. FusionCatcher, SOAPfuse, and JAFFA software were used to identify fusion transcripts from the mRNA sequencing data. Variant calling on the mRNA-seq data was based on the Broad Institute GATK best practices. Gene expression data were generated from the STAR (v2.7.3a)-aligned BAM file using featureCounts and normalized with edgeR. Sub-types were assigned according to identified gene fusions, single nucleotide variants, and gene expression profiles. Gene deletions were detected by multiplex ligation-dependent probe amplification (MLPA). Age-matched ALL patients assessed for in-depth analysis included *P2RY8::CRLF2* (*n* = 38) and *BCR::ABL1* (*n* = 38). Of the *P2RY8::CRLF2* patients, eight harbored +21, two of which were +21c. Four patients harboring *BCR::ABL1* also harbored +21, one of which was +21c (**Supplementary Table S1**).

### 2.12 Multiplex ligation-dependent probe amplification

SALSA MLPA assays #P202, #P335, and #P327 (MRC Holland, Amsterdam, The Netherlands) were performed according to the manufacturer’s instructions using 100 ng of patient DNA and run on a SeqStudio Genetic Analyzer (Applied Biosystems, Waltham, MA, USA).

### 2.13 Quantification and statistical analysis

GraphPad Prism software Version 8.4.0^©^ (GraphPad Software Inc.) and FlowJo software (FlowJo LLC) were used for the analyses. Graphs represent the median value or mean with SEM error bars as indicated in the figure legends. Student’s *t*-test and Welch’s ANOVA were used to determine differences between experimental groups as indicated. Differences were considered statistically significant when the *p*-value was <0.05. Experiments were carried out a minimum of three times (*n* = 3) unless otherwise stated.

## 3 Results

**Figure 1:**
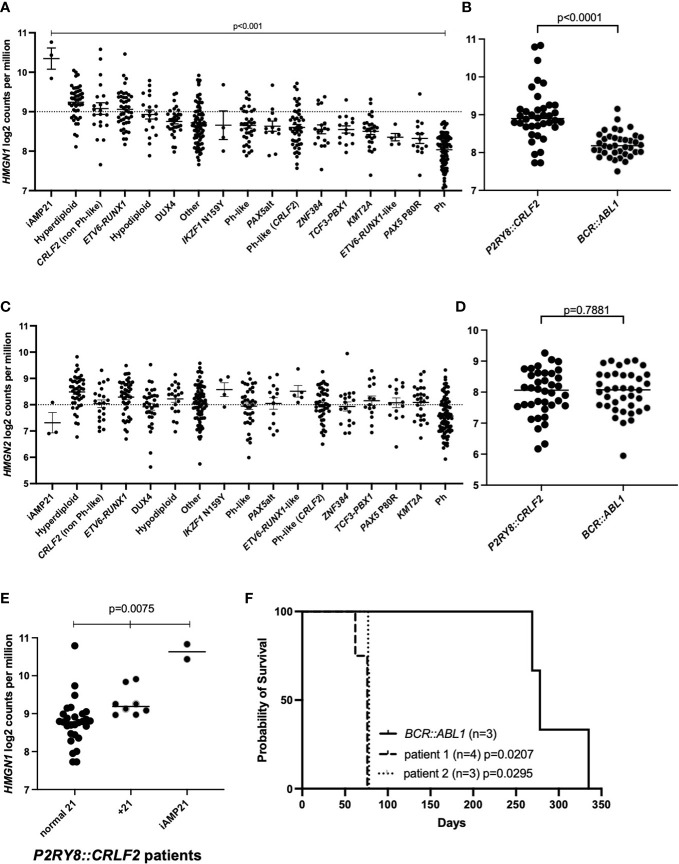
Acute lymphoblastic leukemia (ALL) cohort analysis of high-mobility group nucleosome-binding protein 1 (*HMGN1*) expression. **(A, C)** Gene expression analysis of *HMGN1* or *HMGN2* divided into distinct ALL subtypes. **(B, D)** *HMGN1* or *HMGN2* RNA expression data from 38 age-matched pediatric and adolescent/young adult patients in the purinergic receptor and cytokine receptor-like factor-2 (*P2RY8::CRLF2*) and *BCR::ABL1* control cohorts. Welch’s ANOVA was used to determine significance. **(E)** *HMGN1* expression of *P2RY8::CRLF2* patients divided into normal chromosome 21, + 21, or intrachromosomal amplification of chromosome 21 (iAMP21) groups. **(F)** Kaplan–Meier curve of sub-lethally irradiated NOD.Cg-Prkdc^scid^Il2rg^tm1Wjl^/SzJ (NSG) mice engrafted with *P2RY8::CRLF2* or *BCR::ABL1* leukemia patient cells, analyzed using a log-rank test.

We have assessed a cohort of 580 pediatric and adolescent/young adult B-ALL patients for *HMGN1/2* expression levels. The expression of *HMGN1* varied significantly in ALL patients studied (**Figure 1A**; *p* < 0.001). From these samples, an age-matched cohort of *P2RY8::CRLF2* (*n* = 38) and *BCR::ABL1* (*n* = 38) patients were compared for chromosome 21 alterations and the impact on *HMGN1* expression (**Supplementary Table S1**). A +21 cytogenetic aberration was observed in eight patients with the *P2RY8::CRLF2* gene fusion, two of which were +21c, compared with four patients with +21 who harbored *BCR::ABL1*, one of which was +21c. Two patients with *P2RY8::CRLF2* also harbored iAMP21, whereas no iAMP21 was observed in the *BCR::ABL1* cohort. Significantly higher *HMGN1* expression was identified in patients harboring *P2RY8::CRLF2* compared with the age-matched control *BCR::ABL1*+ ALL patients (**Figure 1B**; *p* < 0.0001). *BCR::ABL1*+ patients were chosen as the control due to their similar gene signature and the number of aged-matched patients to the *P2RY8::CRLF2* group. There was no difference in the expression of the control genes *HMGN2* (*p* = 0.7881) or *JAK2* (*p* = 0.1171) between the cohort of ALL patients or specifically between *P2RY8::CRLF2* and *BCR::ABL1*+ patients (**Figures 1C, D**, **Supplementary Figure S1**). Furthermore, 21% (8/38) of pediatric *P2RY8::CRLF2* ALL patients harbored +21 (*n* = 2 + 21c), resulting in a significantly increased *HMGN1* expression, and 5% (2/38) harbored iAMP21, also with a significantly higher *HMGN1* expression (**Figure 1E**; *p* = 0.0075).

To investigate the latency of patient blasts harboring *P2RY8::CRLF2* and high *HMGN1* expression, NOD.Cg-Prkdc^scid^Il2rg^tm1Wjl^/SzJ (NSG) mice were engrafted with blasts from two ALL patients with *P2RY8::CRLF2* and high *HMGN1* expression (+21). The patient-derived xenograft (PDX) mice succumbed to their leukemia at a median of 76 and 78 days (**Figure 1F**; *p* = 0.0207 and *p* = 0.0295, respectively, compared with the *BCR::ABL1* control with a median survival of 278 days). In addition to *P2RY8::CRLF2*, patient 1 also harbored an activating *JAK2* p.F694L mutation, and patient 2 harbored additional lesions including deletions of *CDKN2A/B* and *IKZF1* exons 4–6 and a *KRAS* p.G12S mutation; however, both patient samples engrafted into NSG mice at the same rate.

**Figure 2:**
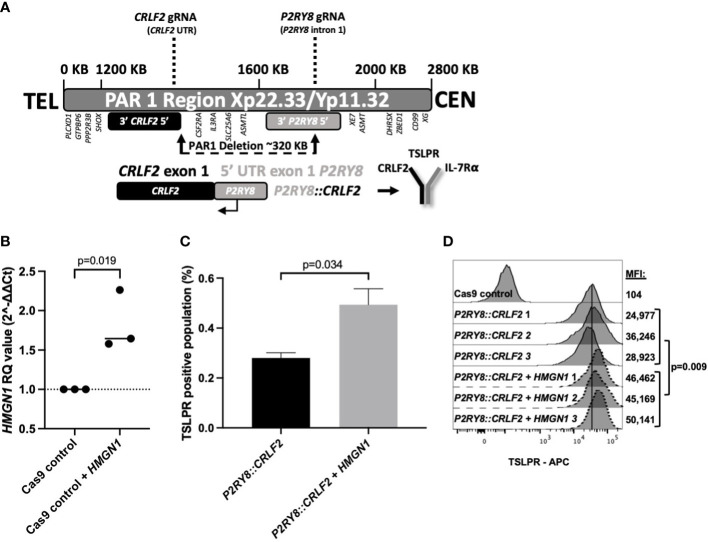
Generating CRISPR/Cas9-edited purinergic receptor and cytokine receptor-like factor-2 (*P2RY8::CRLF2*)-expressing cells and evaluation of functional changes. **(A)** Schematic display of gRNAs, designed using Benchling, targeting the intron after the first non-coding exon of *P2RY8* and the 5′ untranslated region (UTR) of *CRLF2* to create the *P2RY8::CRLF2* breakpoint found in patients. **(B)** Using qRT-PCR to measure high-mobility group nucleosome-binding protein 1 (*HMGN1*) mRNA expression in Jurkat CRISPR/Cas9 cell lines before gRNA transduction. Relative quantification (RQ) values were determined using the housekeeping actin expression and normalized to the parental Cas9 control cells. Student’s *t*-test was used between the *HMGN1* line compared with control Cas9 cells to determine significance. **(C)** Jurkat *P2RY8::CRLF2* cells with or without *HMGN1* expression were stained with thymic stromal lymphopoietin receptor (TSLPR) for flow cytometry after 3 days of gRNA induction to assess the efficiency of *P2RY8::CRLF2* generation and favored repair outcomes. **(D)** TSLPR expression of single-cell clones of Jurkat CRISPR/Cas9-edited *P2RY8::CRLF2* cells measured by flow cytometry.

To further explore the increased *HMGN1* expression and decreased survival in PDX mice, a cell model endogenously expressing *P2RY8::CRLF2* was created with CRISPR/Cas9 to delete 320 kb in the pseudoautosomal region (PAR1) of the X/Y chromosome (**Figure 2A**, **Supplementary Figure S2**). Prior to the induction of *P2RY8* and *CRLF2* gRNAs, *HMGN1* was overexpressed (1.5-fold) in control Cas9-only expressing cells to represent a trisomic level of expression (**Figure 2B**; *p* = 0.019).

Cells with or without *HMGN1* expression were subjected to the same gRNAs/Cas9 without directed repair to determine the favored repair outcome after a DNA break, using TSLPR surface expression as a first readout of successful *P2RY8::CRLF2* fusion creation. *HMGN1*-expressing cells favored *P2RY8::CRLF2* generation as demonstrated by upregulated TSLPR on the cell surface from 0.28% in *P2RY8::CRLF2* cells to 0.49% in cells expressing *P2RY8::CRLF2* + *HMGN1* (**Figure 2C**; *p* = 0.034). This finding suggests that a higher *HMGN1* expression increases the likelihood of *P2RY8::CRLF2* development after a DNA break compared with cells that do not express *HMGN1*-favoring repair for WT *CRLF2*. Upon induction of Cas9 cleavage at *P2RY8* intron 1 and the 5′ UTR of *CRLF2*, cells with and without *HMGN1* expression demonstrated TSLPR surface expression; however, *P2RY8::CRLF2* + *HMGN1* clones had a significantly higher TSLPR expression (MFI: 47,247 ± 1,489; compared with *P2RY8::CRLF2* MFI: 30,049 ± 3,301; *p* = 0.009; **Figure 2D**). The breakpoint PCR of genomic DNA confirmed the presence of the *P2RY8::CRLF2* fusion in a polyclonal pool and single-cell clones of CRISPR/Cas9-edited TSLPR+ cells, consistent with the independent development of *P2RY8::CRLF2* (**Supplementary Figure S2**).

**Figure 3:**
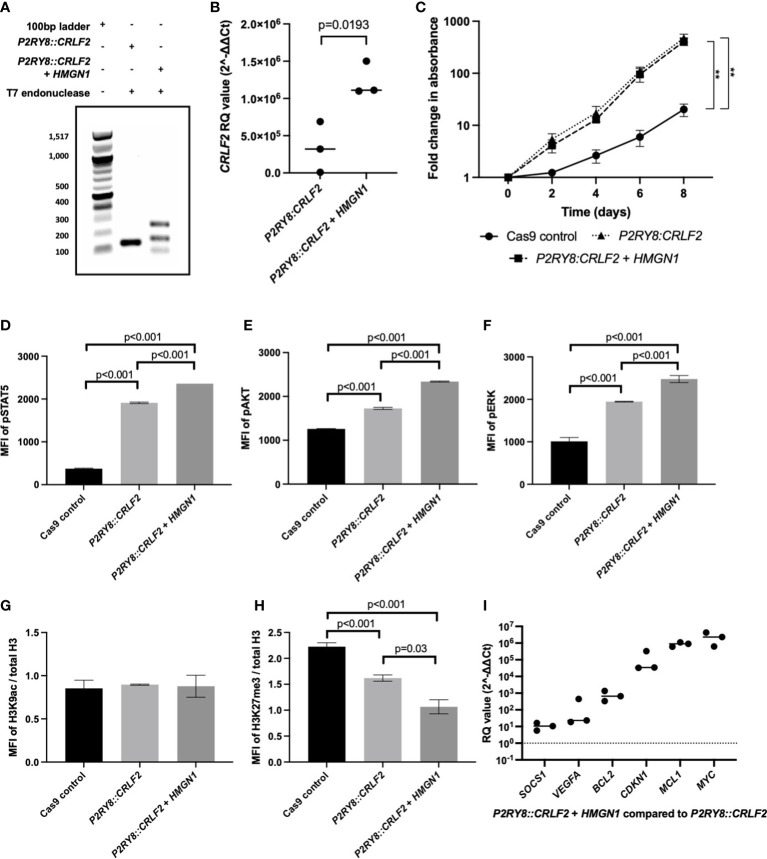
Assessing the effect of high-mobility group nucleosome-binding protein 1 (*HMGN1*) expression on CRISPR/Cas9-edited purinergic receptor and cytokine receptor-like factor-2 (*P2RY8::CRLF2*) cells. **(A)** T7 endonuclease gene editing analysis identifies additional *P2RY8::CRLF2* breakpoint PCR products present in *HMGN1*-expressing cells. **(B)** Using qRT-PCR to measure *CRLF2* mRNA expression in Jurkat CRISPR/Cas9-edited *P2RY8::CRLF2* cell lines. Relative quantification (RQ) values were determined using the housekeeping actin expression and normalized to the parental Cas9 control cells. **(C)** The fold change in the proliferation of Jurkat CRISPR/Cass9-edited *P2RY8::CRLF2* cells was measured over a period of 6 days. Phosphorylation levels of STAT5 **(D)**, AKT **(E)**, ERK **(F)**, or H3K9ac **(G)** and H3K27me3 **(H)** of CRISPR/Cas9-edited *P2RY8::CRLF2* cells with or without high *HMGN1* expression measured by flow cytometry. **(I)** Measuring the expression of genes downstream of STAT5 by qRT-PCR in Jurkat CRISPR/Cas9-edited *P2RY8::CRLF2* cell lines. RQ values were determined using the housekeeping actin expression and normalized to the parental Cas9 control cells. The graphs represent the mean of biological replicates of *n* = 3 with SEM error bars, and a Student’s *t*-test was used between each *P2RY8::CRLF2* cell line compared with control Cas9 cells to determine significance (**p < 0.01).

*HMGN1* may assist in the repair of the double-strand DNA breaks to promote *P2RY8::CRLF2* formation. To confirm this *in vitro*, Cas9 gene editing activity was quantified with or without *HMGN1* expression using a T7-endonuclease assay. The *P2RY8*::*CRLF2* line resulted in only one isoform after T7-endonuclease digestion, whereas the co-expressing *P2RY8::CRLF2* + *HMGN1* line resulted in increased gene editing with three bands present in the population (**Figure 3A**). Sequencing of these isoforms revealed the canonical breakpoint, intron retention, and partial *CRLF2* exon 1 deletion.

Consistent with increased *P2RY8::CRLF2* generation in the *+HMGN1* line, an increase in *CRLF2* mRNA expression was also identified *via* qRT-PCR. *P2RY8::CRLF2* cells had higher *CRLF2* expression [relative quantification (RQ): 3.4 × 10^5^ ± 1.9 × 10^5^] compared with the control cells (RQ: 47 ± 10). Importantly, a significant increase in *CRLF2* expression was observed in *P2RY8::CRLF2* + *HMGN1* cells (RQ: 1.2 × 10^6^ ± 1.3 × 10^5^; *p* < 0.001; **Figure 3B**). The *P2RY8::CRLF2* cell pool grew at a seven-fold higher rate than the Cas9 control cells (**Figure 3C**; *p* < 0.001). This was also observed in the single-cell clones (#1: *p* = 0.005; #2: *p* = 0.015; **Supplementary Figure S3A**). Interestingly, no difference in proliferation was observed between *P2RY8::CRLF2* populations with or without *HMGN1* expression despite the increase in *CRLF2* expression, suggesting that the role of *HMGN1* is epigenetic rather than a direct effect on cell proliferation. 

To determine the effect of HMGN1 on cell signaling, the phosphorylation (p) levels of STAT5, AKT, and ERK were assessed. Interestingly, a stepwise increase in phosphorylation of all three proteins was observed between the Cas9 control cells (MFI of pSTAT5: 373 ± 7.4; pAKT: 1,258 ± 5; pERK: 1,011 ± 52), the *P2RY8::CRLF2* cells (MFI of pSTAT5: 1,910 ± 10.2; pAKT: 1,727 ± 13.5; pERK: 1,946 ± 6.3), and the *P2RY8::CRLF2* + *HMGN1* cells (MFI of pSTAT5: 2,359 ± 1; pAKT: 2,339.6 ± 6.3; pERK: 2,478 ± 47.5; **Figures 3D–F**; all *p* < 0.001). This signaling profile is consistent with the reported phenotype of *P2RY8::CRLF2* patients.

As HMGN1 is a demethylase, the acetylation of H3K9 and trimethylation of H3K27 were assessed. No change in gene activation was identified (**Figure 3G**); however, a stepwise decrease in H3K27me3 was identified from Cas9 control cells (**Figure 3H**; H3K27me3 MFI: 2.2 ± 0.04) to *P2RY8::CRLF2* cells (H3K27me3 MFI: 1.6 ± 0.03; *p* < 0.001), with a further reduction in *P2RY8::CRLF2* + *HMGN1* cells (H3K27me3 MFI: 1 ± 0.07; *p* = 0.03). This reduction in H3K27me3 may indicate that previously silenced genes have become active in this line.

Assessment of pSTAT5-mediated transcriptional activation indicated higher expression levels of *BCL2*, *CDKN1*, and particularly *MCL1* and *MYC* in cells co-expressing *HMGN1* and *P2RY8::CRLF2* compared with Cas9 control cells (**Figure 3I**). This finding suggests the potential leukemic survival mechanisms in ALL patients with an increased expression of *HMGN1* and the *P2RY8::CRLF2* fusion.

## 4 Discussion

We have evaluated an Australian cohort of pediatric/adolescent B-ALL patients and identified a significantly higher *HMGN1* expression in *P2RY8::CRLF2* ALL patients compared with a control subgroup. In particular, a significantly higher *HMGN1* expression was observed in *P2RY8::CRLF2* patients with +21 or iAMP21. In a PDX model of two separate patients with *P2RY8::CRLF2* and high *HMGN1* expression, the mice succumbed to the disease at the same rate, indicating an aggressive disease burden despite the additional lesions in each patient’s blasts. This was compared with the engraftment of blasts from a patient with *BCR::ABL1* which had a latency 3.6 times slower than the *P2RY8::CRLF2* blasts. To test the hypothesis that *HMGN1* is an influencing factor for *P2RY8::CRLF2* development, a cell line in a state before *P2RY8::CRLF2* development was required.

Modeling loss-of-function tumor suppressors or gain-of-function oncogenes is fundamental to studying cancer, and CRISPR/Cas9 technology has streamlined this process with high efficiency. Recurrent chromosomal alterations and novel gene fusions have been and continue to be identified in ALL patients (30). To understand the implications of these alterations, they need to be modeled using *in vitro* and *in vivo* systems. Subsequently, mechanistic assays and drug panels can be used to identify therapeutic candidates to rationally target the leukemic cells harboring these lesions. The current modeling of ALL gene fusions involves cloning, which can be complex with repetitive sequences or very large transcripts. CRISPR/Cas9 presents a solution to overcome these difficulties and has been used to create chromosomal alterations found in other diseases (24, 26, 31) but has not been previously attempted in ALL. We have utilized this technology to generate an inducible endogenous cell model of *P2RY8::CRLF2* that can be employed to determine co-occurring factors in leukemogenesis.

The *P2RY8::CRLF2* fusion alone is not sufficient for leukemic transformation and frequently co-occurs with mutations in Janus kinase 2 (*JAK2*) (7). *P2RY8::CRLF2* is increased in DS-ALL patients with a frequency of ~60% (8); however, these patients do not harbor *JAK* mutations as frequently as non-DS-ALL patients (32). Therefore, the “double hit” is likely to occur first on chromosome 21 and remains to be identified. We have previously demonstrated that a frameshift mutation in *NF1* can cooperate with *P2RY8::CRLF2* as a mechanism of leukemic relapse (33). Therefore, patients with *P2RY8::CRLF2* who do not harbor a JAK or Ras pathway mutation need to be carefully assessed to determine the cooperating lesions for leukemic transformation. Previous reports have demonstrated that increased *HMGN1* expression results in a B-cell progenitor phenotype due to its role in lineage determination (11) and may cooperate with *P2RY8::CRLF2*.

The CRISPR/Cas9 model generated here allowed a pre-leukemic state to be modeled. Increasing the *HMGN1* expression before inducing *P2RY8* and *CRLF2* gRNAs favored fusion development. A potential mechanism *via* increased DNA double-strand break repair was demonstrated. Consistent with this role of DNA repair, previous reports have identified that the loss of *HMGN1* leads to an impaired DNA damage response (34). Therefore, in a trisomy 21 cell with increased *HMGN1*, an increased chance of repairing double-strand breaks to create *P2RY8::CRLF2* is likely. This finding indicates that *HMGN1* expression may increase the susceptibility of *P2RY8::CRLF2* development.

The *P2RY8::CRLF2* and *HMGN1* co-expressing cells demonstrated increased proliferation and TSLPR expression and had the most clinically relevant trends in cell signaling compared with previous reports of *CRLF2* patient cell signaling (35). Therefore, using CRISPR/Cas9, a role for *HMGN1* in cooperation with *P2RY8::CRLF2* was demonstrated. Interestingly, *P2RY8::CRLF2* + *HMGN1* cells had increased expression of *BCL2*, *CDKN1A*, *MCL1*, and *MYC*. Furthermore, a global decrease in H3K27me3 was associated with an increase in transcriptional activation, consistent with previous reports (11). Increased *MYC* expression has been previously demonstrated in *HMGN1*-overexpressing cells (11) and trisomy 21 B-ALL cells (36). BCL2 and MCL1 are anti-apoptotic and novel targets in ALL that warrant further investigation in DS-ALL patients.

The *P2RY8::CRLF2* gene fusion is prevalent in DS, +21, and iAMP21 ALL patients. Here we demonstrate that *P2RY8::CRLF2* is associated with a high expression of *HMGN1* (chr21) in ALL patient cells. Using CRISPR/Cas9 in an *in vitro* model, we demonstrate that forced high expression of *HMGN1* alters the DSB repair mechanism, favoring PAR1 deletion and the subsequent formation of the *P2RY8::CRLF2* gene fusion (7), with associated higher expression of STAT5 target genes. Furthermore, this was achieved by the first reported CRISPR/Cas9 320-kb deletion resulting in a clinically relevant fusion gene found in ALL. Importantly, this model will be valuable to advance the ALL field by investigating leukemia-initiating events as the inducible gRNAs allow recapitulation of a pre-leukemic state. Understanding the role of *HMGN1* in the disproportionate number of DS–ALL patients who are diagnosed with *P2RY8::CRLF2* ALL has the potential to lead to novel therapeutic interventions in this high-risk group of patients where effective therapeutic options are currently limited.

The limitations of this study include the use of viral vectors to deliver the Cas9 machinery into the cell of interest rather than a transient expression system. This was necessary to overcome the low efficiency of transfection in leukemic cells; however, we recognize that this could potentially lead to the disruption of oncogenes at any given locus. Additionally, the Cas9 system has been reported to sporadically induce large deletions in a chromosome (37). In this case, it was a large deletion that was directed by the Cas9 machinery to result in the *P2RY8::CRLF2* gene fusion but this does not infer that other deletions did not occur. RNA sequencing could be utilized to determine if any other chromosomal regions were disrupted. To validate the specificity of *HMGN1* susceptibility to *P2RY8::CRLF2*, this study could be repeated to model another gene fusion with the same approach, such as *ETV6::RUNX1* which is present in ~10% of DS–ALL patients (38).

## Data availability statement

Additional data and requests for resources should be directed to the lead contact, Deborah White (deborah.white@sahmri.com). Materials can be obtained via material transfer agreement from authors’ institutions upon reasonable request to corresponding authors.

## Ethics statement

Studies involving human participants were reviewed and approved by each relevant institutional Human Research Ethics Committee. Written informed consent to participate in this study was provided by the participants’ legal guardian/next of kin. Animal studies were reviewed and approved by the South Australian Health and Medical Research Institute Ethics Committee.

## Author contributions

EP, SH, and DW conceived of and designed the experiments. PT, DY, and DW provided all study materials. ECP collected, assembled, and analyzed the data and wrote the manuscript. JR performed the bioinformatics analysis. DW, SH, PT, DY, and JR critically revised the manuscript. All authors contributed to the article and approved the submitted version.
