# Supplementary material for: Gain of chromosome 21 increases the propensity for P2RY8: :CRLF2 acute lymphoblastic leukemia via increased HMGN1 expression
Source: Front Oncol. 2023 Jul 6;13:1177871. doi: 10.3389/fonc.2023.1177871 (PMC10358767; doi:10.3389/fonc.2023.1177871)
Supplement: Supplementary file 1 [file DataSheet_1.pdf]

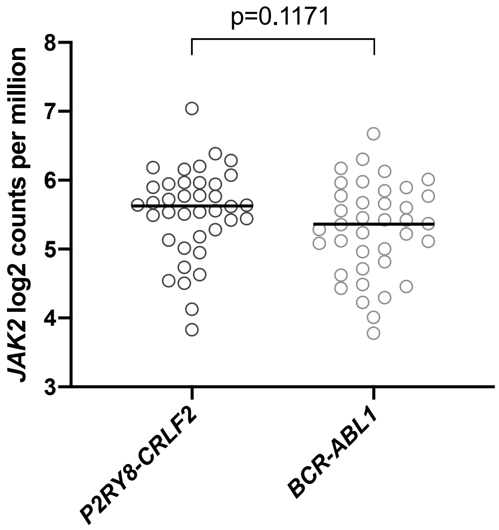

Supplementary Figure 1: *JAK2* gene expression from mRNA sequencing. and *JAK2* RNA expression data from 38 age-matched pediatric patients in the *P2RY8::CRLF2* and control *BCR-ABL1* cohorts. A Welch's ANOVA was used to determine significance.

A ***P2RY8::CRLF2* pool gDNA breakpoint**

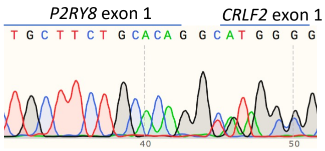

B ***P2RY8::CRLF2* cDNA breakpoint**

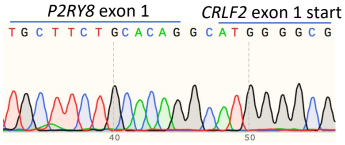

C ***P2RY8::CRLF2 HMGN1* gDNA breakpoint**

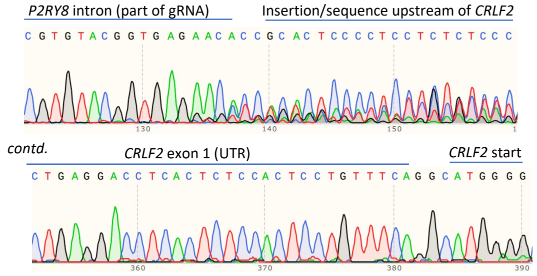

Supplementary Figure 2: Generating CRISPR/Cas9 edited *P2RY8::CRLF2* expressing cells and evaluation of breakpoints. PCR amplification from gDNA and cDNA of the *P2RY8::CRLF2* breakpoint in a pool of CRISPR/Cas9 edited *P2RY8::CRLF2* cells. Sanger sequencing of pooled populations of CRISPR/Cas9 edited *P2RY8::CRLF2* cells **(A-B)** and *P2RY8::CRLF2* + *HMGN1* cells **(C)**.

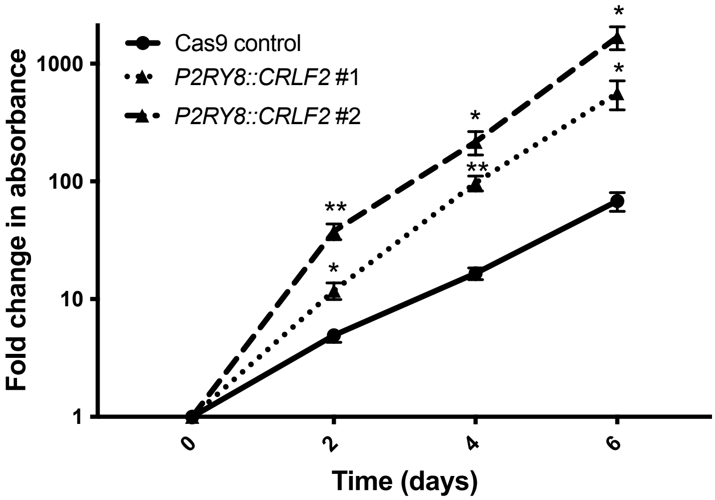

Supplementary Figure 3: Characterising the proliferation of *P2RY8::CRLF2* cells. A) The fold change in proliferation of CRISPR/Cas9 edited *P2RY8::CRLF2* single cell clones measured over a period of 6 days. The graph represents the mean of biological replicate of n=3 with SEM error bars and a student's *t*-test was used between the Cas9 cell line and each *P2RY8::CRLF2* expressing line to determine significance, $^{*}p<0.05$, $^{**}p<0.01$, $^{***}p<0.001$.
