# Supplementary material for: Gain of chromosome 21 increases the propensity for P2RY8: :CRLF2 acute lymphoblastic leukemia via increased HMGN1 expression
Source: Front Oncol. 2023 Jul 6;13:1177871. doi: 10.3389/fonc.2023.1177871 (PMC10358767; doi:10.3389/fonc.2023.1177871)
Supplement: Supplementary file 2 [file DataSheet_2.pdf]

Table SI: ALL patient genomic features and *HMGN1* expression level.			
**Patient**	**Subtype**	**HMGN1 log2 CPM**	**Chr 21 status**
ALL01	P2RY8::CRLF2	8.970815689	plus 21
ALL02	P2RY8::CRLF2	9.083487678	plus 21
ALL03	P2RY8::CRLF2	9.836591087	plus 21
ALL04	P2RY8::CRLF2	10.83027124	iAMP21
ALL05	P2RY8::CRLF2	8.357181883	
ALL06	P2RY8::CRLF2	7.728253811	
ALL07	P2RY8::CRLF2	8.881704888	
ALL08	P2RY8::CRLF2	8.777778309	
ALL09	P2RY8::CRLF2	8.793060202	
ALL10	P2RY8::CRLF2	8.914239463	
ALL11	P2RY8::CRLF2	9.909499321	plus 21
ALL12	P2RY8::CRLF2	8.967302647	plus 21
ALL13	P2RY8::CRLF2	8.990856264	
ALL14	P2RY8::CRLF2	8.708941865	
ALL15	P2RY8::CRLF2	8.795016349	
ALL16	P2RY8::CRLF2	8.994606323	
ALL17	P2RY8::CRLF2	8.847961006	
ALL18	P2RY8::CRLF2	9.25431388	plus 21
ALL19	P2RY8::CRLF2	10.43479848	iAMP21
ALL20	P2RY8::CRLF2	9.052010685	
ALL21	P2RY8::CRLF2	7.729348254	
ALL22	P2RY8::CRLF2	9.124377526	constitutive +21
ALL23	P2RY8::CRLF2	8.67814616	
ALL24	P2RY8::CRLF2	9.143860974	
ALL25	P2RY8::CRLF2	8.44300725	
ALL26	P2RY8::CRLF2	9.164297707	
ALL27	P2RY8::CRLF2	8.695615063	
ALL28	P2RY8::CRLF2	8.762628569	
ALL29	P2RY8::CRLF2	9.481050791	
ALL30	P2RY8::CRLF2	8.807645469	
ALL31	P2RY8::CRLF2	8.004437236	
ALL32	P2RY8::CRLF2	9.25656127	constitutive +21
ALL33	P2RY8::CRLF2	8.670023808	
ALL34	P2RY8::CRLF2	10.78897932	
ALL35	P2RY8::CRLF2	9.733334206	
ALL36	P2RY8::CRLF2	8.474351359	
ALL37	P2RY8::CRLF2	8.280574904	
ALL38	P2RY8::CRLF2	7.951454267	
ALL39	BCR::ABL1	8.449093057	
ALL40	BCR::ABL1	8.358624755	
ALL41	BCR::ABL1	8.062752264	
ALL42	BCR::ABL1	8.125859267	
ALL43	BCR::ABL1	8.598292823	
ALL44	BCR::ABL1	8.037185034	
ALL45	BCR::ABL1	8.176724557	
ALL46	BCR::ABL1	8.59550281	plus 21
ALL47	BCR::ABL1	8.141971069	
ALL48	BCR::ABL1	7.503763734	
ALL49	BCR::ABL1	7.957320819	constitutive +21
ALL50	BCR::ABL1	8.182479573	
ALL51	BCR::ABL1	8.312127222	
ALL52	BCR::ABL1	7.895753722	
ALL53	BCR::ABL1	7.849430151	
ALL54	BCR::ABL1	8.001293145	
ALL55	BCR::ABL1	8.012726304	
ALL56	BCR::ABL1	8.200927936	
ALL57	BCR::ABL1	7.875937099	
ALL58	BCR::ABL1	7.755074802	
ALL59	BCR::ABL1	7.799729858	
ALL60	BCR::ABL1	8.148121621	
ALL61	BCR::ABL1	8.25180109	
ALL62	BCR::ABL1	8.878911943	
ALL63	BCR::ABL1	7.860616874	
ALL64	BCR::ABL1	8.627896015	
ALL65	BCR::ABL1	8.67775371	
ALL66	BCR::ABL1	8.424616354	
ALL67	BCR::ABL1	8.475484081	
ALL68	BCR::ABL1	7.995808116	
ALL69	BCR::ABL1	8.351008074	
ALL70	BCR::ABL1	8.01925799	
ALL71	BCR::ABL1	8.405607916	plus 21
ALL72	BCR::ABL1	8.384301013	
ALL73	BCR::ABL1	8.069346258	
ALL74	BCR::ABL1	8.275139889	
ALL75	BCR::ABL1	9.155090462	plus 21
ALL76	BCR::ABL1	8.331439912	
