# Supplementary material for: Gain of chromosome 21 increases the propensity for P2RY8: :CRLF2 acute lymphoblastic leukemia via increased HMGN1 expression
Source: Front Oncol. 2023 Jul 6;13:1177871. doi: 10.3389/fonc.2023.1177871 (PMC10358767; doi:10.3389/fonc.2023.1177871)
Supplement: Supplementary file 3 [file DataSheet_3.pdf]

Key Resources Table

REAGENT or RESOURCE	SOURCE	IDENTIFIER
**Antibodies**		
TSLPR-APC	Invitrogen	Cat#17-5499-41
IgG2a-APC	Invitrogen	Cat#17-4724-81
IgG2a-PE	BD	Cat#556653
IgG1-APC	BD	Cat#551019
pSTAT5-PE	BD	Cat#612567
pERK-PE	BD	Cat#665426
pS6 kinase	Cell Signaling Technologies	Cat#665426
pAKT	BD	Cat#560378
H3K9ac	Cell Signaling Technologies	Cat#4484
H3K27me3	Cell Signaling Technologies	Cat#12158
**Bacterial and Virus Strains**		
FuCas9mCherry	Addgene	Cat#70182
FgH1tUTG	Addgene	Cat#70183
pMD2.G	Addgene	Cat#12259
pMDL-PRRE	Addgene	Cat#12251
pRSV-REV	Addgene	Cat#12253
pMImCII HMGN1	This paper	
**Chemicals, Peptides, and Recombinant Proteins**		
Doxycycline Hyclate	Sigma-Aldrich	Cat#D9891
Esp3I	New England Biolabs	Cat#R0734L
rSAP	New England Biolabs	Cat#M0371L
T4 PNK	New England Biolabs	Cat#M0201L
T4 Ligase	New England Biolabs	Cat#M0204L
Lipofectamine 2000	Invitrogen	Cat#11668-019
Polybrene	Merck	Cat#TR-1003-G
TRIzol	Invitrogen	Cat#15596026
T7 endonuclease	New England Biolabs	Cat#M0263L
**Critical Commercial Assays**		
CellTiter Glo 2.0	Promega	Cat#G9243
SYBR Green	Qiagen	Cat#330503
PCR Phusion Kit	New England Biolabs	Cat#E0553L
Quantitect Reverse Transcriptase	Qiagen	Cat#205313
QIAquick Gel extraction kit	Qiagen	Cat#28706
**Experimental Models: Cell Lines**		
Jurkat cells	ATCC	(ATCC® TIB-152™)
HEK293T cells	ATCC	(ATCC® CRL-11268™)
**Experimental Models: Organisms/Strains**		
DH5α	New England Biolabs	Cat#12297016

Oligonucleotides		
P2RY8 intron gRNA 5’ -CGTGTACGGTGAGAACATGG- 3’	This Paper	N/A
Pre-*CRLF2* gRNA 5’ -GTGCGTGGCAGTCCTGATCC- 3’	This Paper	N/A
CRLF2 UTR gRNA 5’ -CTGTTCGTTGTAGGTCCCTG- 3’	This Paper	N/A
CRLF2 qPCR F 5’ -TGGATCACAGACACCCAGAA- 3’	This Paper	N/A
CRLF2 qPCR R 5’ -TCTTGGCCAACTGGACTACC- 3’	This Paper	N/A
HMGN1 qPCR F 5’ -TGCAAACAAAAGGGAAAAGG- 3’	This Paper	N/A
HMGN1 qPCR R 5’ -CATCAGAGGCTGGACTCTCC- 3’	This Paper	N/A
P2RY8 seq 5’ -AAGCGTTGCATCCTGTTACCTGG- 3’	This Paper	N/A
CRLF2 seq 5’ -GCCTCCCAGCAGAAAGACGG- 3’	This Paper	N/A
*VEGFA*_qPCR_F 5’ –AGCACAGCAGATGTGAATGC- 3’	This Paper	N/A
*VEGFA*_qPCR_R 5’ –TTTCTTGCGCTTTCGTTTTT- 3’	This Paper	N/A
*BCL2*_qPCR_F 5’ -AAGCTGTCACAGAGGGGCTA- 3’	This Paper	N/A
*BCL2*_qPCR_R 5’ -CAGGCTGGAAGGAGAAGATG- 3’	This Paper	N/A
*MCL1*_qPCR_F 5’ -GCTCCGGAAACTGGACATTA- 3’	This Paper	N/A
*MCL1*_qPCR_R 5’ -CCCAGTTTGTTACGCCATCT- 3’	This Paper	N/A
*MYC*_qPCR_F 5’ -CCAGATCCCTGAATTGGAAA- 3’	This Paper	N/A
*MYC*_qPCR_R 5’ -TCGTCTGCTTGAATGGACAG- 3’	This Paper	N/A
*GATA3*_qPCR_F 5’ -CTTATCAAGCCCAAGCGAAG- 3’	This Paper	N/A
*GATA3*_qPCR_R 5’ -CATTAGCGTTCCTCCTCCAG- 3’	This Paper	N/A
*CDKN1A*_qPCR_F 5’ -CGGTGGAACTTTGACTTCGT- 3’	This Paper	N/A
*CDKN1A*_qPCR_R 5’ -CAGGGCAGAGGAAGTACTGG- 3’	This Paper	N/A
*SOCS1*_qPCR_F 5’ -CCTCCTCGTCCTCGTCTTC- 3’	This Paper	N/A
*SOCS1*_qPCR_R 5’ -AAGGTGCGGAAGTGAGTGTC- 3’	This Paper	N/A
**Software and Algorithms**		
Prism version 8.4.0	GraphPad	https://www.graphpad.com
FlowJo	FlowJo, LLC	https://www.flowjo.com/solutions/
Benchling	Biology Software, 2019	https://benchling.com
